# 1-(4-Ethoxy­benzo­yl)-4-(4-methoxy­phen­yl)thiosemicarbazide

**DOI:** 10.1107/S1600536809036599

**Published:** 2009-10-03

**Authors:** Saira Khanum, Muhammad Farman, Nasim H. Rama, Shahid Hameed, Peter G. Jones

**Affiliations:** a Department of Chemistry, Quaid-i-Azam University, Islamabad-45320, Pakistan; b Institut für Anorganische und Analytische Chemie, Technische Universität Braunschweig, Postfach 3329, 38023 Braunschweig, Germany

## Abstract

The title compound, C_17_H_19_N_3_O_3_S, crystallizes with two closely similar independent mol­ecules related by a pseudotranslation of **c**/2. Each mol­ecule consists of three approximately planar moieties centred on the N_2_CS group and the two ring systems. The packing involves classical H bonds of the form N_amide_—H⋯S and N_hydrazine_—H⋯OC, together with various weak hydrogen bonds and N_hydrazine_—H⋯π inter­actions. The overall packing is three-dimensional, but layer substructures parallel to the *xz* plane can be readily identified. Each mol­ecule forms a topologically equivalent set of hydrogen-bond inter­actions.

## Related literature

Thio­semicarbazides represent a class of versatile precursors for the syntheses of various nitro­gen heterocycles, see: Al-Masoudi *et al.* (2006 ▶); Kucukguzel *et al.* (2007 ▶); Serwar *et al.*2009 ▶); Serwer *et al.* (2008 ▶); Tomascikava *et al.* (2008 ▶); Tozkoparan *et al.* (2007 ▶). For the pharmaceutical potential of the thio­semicarbazide moiety, see: Angelusiu *et al.* (2009 ▶); Ghosh *et al.* (2009 ▶); Liu *et al.* (2009 ▶).
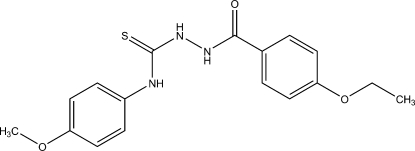


## Experimental

### 

#### Crystal data

C_17_H_19_N_3_O_3_S
*M* *_r_* = 345.41
Monoclinic, 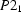

*a* = 15.40429 (13) Å
*b* = 9.67120 (9) Å
*c* = 11.69922 (9) Å
β = 95.0922 (7)°
*V* = 1736.05 (3) Å^3^
*Z* = 4
Mo *K*α radiation
μ = 0.21 mm^−1^
*T* = 100 K
0.3 × 0.2 × 0.1 mm

#### Data collection

Oxford Diffraction Xcalibur E diffractometer
Absorption correction: multi-scan (CrysAlisPro; Oxford Diffraction, 2009 ▶) *T*_min_ = 0.981, *T*_max_ = 1.000
44704 measured reflections
9918 independent reflections
6348 reflections with *I* > 2σ(*I*)
*R*_int_ = 0.032

#### Refinement

*R*[*F* ^2^ > 2σ(*F* ^2^)] = 0.029
*wR*(*F* ^2^) = 0.055
*S* = 0.96
9918 reflections
461 parameters
16 restraints
H atoms treated by a mixture of independent and constrained refinement
Δρ_max_ = 0.30 e Å^−3^
Δρ_min_ = −0.23 e Å^−3^
Absolute structure: Flack (1983 ▶), 4562 Friedel pairs
Flack parameter: 0.01 (3)

### 

Data collection: *CrysAlisPro* (Oxford Diffraction, 2009 ▶); cell refinement: *CrysAlisPro*; data reduction: *CrysAlisPro*; program(s) used to solve structure: *SHELXS97* (Sheldrick, 2008 ▶); program(s) used to refine structure: *SHELXL97* (Sheldrick, 2008 ▶); molecular graphics: *XP* (Siemens, 1994 ▶); software used to prepare material for publication: *SHELXL97*.

## Supplementary Material

Crystal structure: contains datablocks global, I. DOI: 10.1107/S1600536809036599/bt5057sup1.cif

Structure factors: contains datablocks I. DOI: 10.1107/S1600536809036599/bt5057Isup2.hkl

Additional supplementary materials:  crystallographic information; 3D view; checkCIF report

## Figures and Tables

**Table 1:** Hydrogen-bond geometry (Å, °)

*D*—H⋯*A*	*D*—H	H⋯*A*	*D*⋯*A*	*D*—H⋯*A*
N1—H01⋯S′	0.826 (11)	2.735 (12)	3.4483 (14)	145.5 (14)
N4—H04⋯O1′^i^	0.812 (12)	2.016 (13)	2.8156 (17)	168.2 (17)
N1′—H01′⋯S^ii^	0.816 (11)	2.659 (12)	3.3772 (14)	147.8 (14)
N4′—H04′⋯O1^i^	0.836 (12)	2.011 (13)	2.8228 (18)	163.7 (15)
C11′—H11′⋯O1^i^	0.95	2.41	3.3378 (19)	166
C11—H11⋯O1′^i^	0.95	2.38	3.2927 (18)	162
C20—H20*B*⋯S^iii^	0.98	2.98	3.9215 (17)	161
C20′—H20*E*⋯S′^iv^	0.98	2.92	3.8936 (18)	175
C10′—H10′⋯S^i^	0.95	2.83	3.7559 (17)	166
C10—H10⋯S′^i^	0.95	2.81	3.7369 (17)	166
C15′—H15′⋯S^ii^	0.95	3.05	3.6482 (16)	123
C15—H15⋯S′	0.95	2.94	3.6925 (17)	137
N3′—H03′⋯*Cg*	0.847 (10)	2.53	3.25	144
N3—H03⋯*Cg*′^v^	0.822 (10)	2.65	3.29	135

Symmetry codes: (i) 
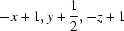
; (ii) 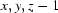; (iii) 
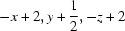
; (iv) 
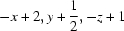
; (v) 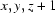. *Cg* is the centroid of the C6–C11 ring and *Cg*′ is the centroid of the C6′–C11′ ring.

**Fig. 1.**
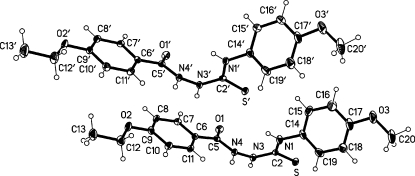
Molecular structure of the title compound showing the two independent molecules, the atom labelling scheme and displacement ellipsoids at the 50% probability level.

**Fig. 2.**
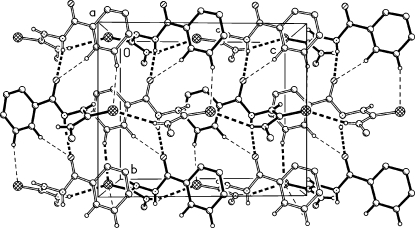
A section of the three-dimensional packing diagram of the title compound viewed parallel to the x axis. Bond types: thick continuous bonds, molecule 1; open continuous bonds, molecule 2; thick dashed bonds, classical H bonds; thin dashed bonds, C—H···O interactions. For clarity, the ethoxy substituents and all H atoms not involved in the H bonds were omitted and the rings at N1 are shown only as their ipso carbons.

## supplementary crystallographic information

### Comment

Thiosemicarbazides represent a class of versatile precursors for the syntheses of various nitrogen heterocycles (Kucukguzel *et al.*, 2007; Al-Masoudi *et al.*, 2006; Tozkoparan *et al.*, 2007; Tomascikava *et al.*, 2008; Serwer *et al.* 2008; Serwar *et al.* 2009). The thiosemicarbazide moiety possesses substantial pharmaceutical potential such as anti-tumor (Angelusiu *et al.*, 2009), tyrosinase inhibitor (Liu *et al.*, 2009) and antioxidant (Ghosh *et al.*, 2009) properties. The title compound was synthesized as an intermediate for its onward conversion to 1,2,4-triazoles and 1,3,4-thiadiazoles and in order to explore their anti-bacterial, urease inhibition and anti-fungal activities.

The title compound crystallizes with two independent molecules in the asymmetric unit (Fig. 1); these are related to a good approximation by a translation of c/2. A least-squares fit of both molecules gives an r.m.s. deviation of 0.15 Å for all non-H atoms. Main differences involve orientations of the aromatic rings, *e.g.* C2—N1—C14—C15 131.9 (2), 122.1 (2)°. Atoms of the second independent molecule are distinguished by a prime (') where necessary. Molecular dimensions (*e.g.* the hydrazine N—N bond lengths of 1.393 (2), 1.391 (2) Å) may be regarded as normal. The thione sulfur is *trans* to N4 across the bond C2—N3 (torsion angles 174.8 (1), 172.2 (1)°) but *cis* to C14 across the bond N1—C2 (torsion angles -9.0 (2), -8.1 (2)).

Each molecule consists of three approximately planar moieties with various common atoms: (1) the central thioamide section (S, N2, N3, N4, C2, C14); (2) the hydrazine and its aromatic ring (C5–13, O1, O2, N3, N4); and (3) the amide and its aromatic ring (C14–20, N1, O3). These display r.m.s. deviations of 0.07, 0.05, 0.01 Å in molecule 1 and 0.08, 0.06, 0.01 Å in molecule 2. Interplanar angles (1)–(2) and (3)–(2) are 68.09 (3), 56.56 (4)° in molecule 1 and 77.22 (3), 66.14 (3)° in molecule 2.

The molecular packing is three-dimensional; classical and "weak" hydrogen bonds are summarized in Table 2. Both molecules form a topologically equivalent set of hydrogen bonds. Fig. 2 shows that the molecules associate to form layers parallel to the *xz* plane; within the layers, the molecules are linked by classical H bonds N1—H01···S' (and N1'—H01'···S), whereas the layers are linked by N4—H04···O1' and the weak H bonds C10—H10···S' and C11—H11···O1' (and their counterparts). The H bond donors N3—H03 and N03'—H03' are involved in C—H···π interactions to the centroids of the ring C6'–11' and C6–11 respectively, with H···π 2.65, 2.53 Å and angles 135°, 144° respectively (operators *x*,*y*,1 + *z*, and *x*,*y*,*z*). These interactions are not shown explicitly in Fig. 2 but can be recognized within the layers.

### Experimental

4-Ethoxybenzohydrazide (0.0068 moles) was dissolved in methanol (30 ml) and a solution of 4-methoxyphenylisothiocyanate (0.0066 moles), separately dissolved in 10 ml of methanol, was added dropwise with continuous stirring. The reaction mixture was refluxed for 10–12 h and progress of the reaction monitored by TLC. After consumption of the starting materials, the mixture was cooled to room temperature. The methanol was removed to give the crude thiosemicarbazide as an oil that solidified on cooling. The product was recrystallized from ethanol as large colourless blocks that were cut to size for X-ray analysis.

### Refinement

The NH H atoms were refined freely but with N—H distances restrained equal. Methyl H atoms were identified in difference syntheses, idealized and refined as rigid groups with C—H 0.98 Å and H—C—H angles 109.5°, allowed to rotate but not tip. Other H atoms were placed in calculated positions and refined using a riding model with C—H_arom_ 0.95 and *C*—H_methylene_ 0.99 Å; the hydrogen *U* values were fixed at 1.5 (methyl) or 1.2 × *U*(eq) of the parent carbon atom.

The compound is achiral but crystallizes by chance in a chiral space group. The translational pseudosymmetry causes the reflections with *l* odd to be weak, but they are definitely present.

### Crystal data

**Table:**

C_17_H_19_N_3_O_3_S	*F*(000) = 728
*M**_r_* = 345.41	*D*_x_ = 1.322 Mg m^−^^3^
Monoclinic, *P*2_1_	Mo *K*α radiation, λ = 0.71073 Å
*a* = 15.40429 (13) Å	Cell parameters from 16506 reflections
*b* = 9.67120 (9) Å	θ = 2.3–30.7°
*c* = 11.69922 (9) Å	µ = 0.21 mm^−^^1^
β = 95.0922 (7)°	*T* = 100 K
*V* = 1736.05 (3) Å^3^	Tablet, colourless
*Z* = 4	0.3 × 0.2 × 0.1 mm

### Data collection

**Table:**

Oxford Diffraction Xcalibur E diffractometer	9918 independent reflections
Radiation source: Enhance (Mo) X-ray Source	6348 reflections with *I* > 2σ(*I*)
graphite	*R*_int_ = 0.032
Detector resolution: 16.1419 pixels mm^-1^	θ_max_ = 30.5°, θ_min_ = 2.3°
ω–scan	*h* = −21→21
Absorption correction: multi-scan (*CrysAlis PRO*; Oxford Diffraction, 2009)	*k* = −13→13
*T*_min_ = 0.981, *T*_max_ = 1.000	*l* = −15→16
44704 measured reflections	

### Refinement

**Table:**

Refinement on *F*^2^	Secondary atom site location: difference Fourier map
Least-squares matrix: full	Hydrogen site location: inferred from neighbouring sites
*R*[*F*^2^ > 2σ(*F*^2^)] = 0.029	H atoms treated by a mixture of independent and constrained refinement
*wR*(*F*^2^) = 0.055	*w* = 1/[σ^2^(*F*_o_^2^) + (0.020*P*)^2^] where *P* = (*F*_o_^2^ + 2*F*_c_^2^)/3
*S* = 0.96	(Δ/σ)_max_ = 0.001
9918 reflections	Δρ_max_ = 0.30 e Å^−^^3^
461 parameters	Δρ_min_ = −0.23 e Å^−^^3^
16 restraints	Absolute structure: Flack (1983), 4562 Friedel pairs
Primary atom site location: structure-invariant direct methods	Flack parameter: 0.01 (3)

### Special details

**Table:**

Geometry. All e.s.d.'s (except the e.s.d. in the dihedral angle between two l.s. planes) are estimated using the full covariance matrix. The cell e.s.d.'s are taken into account individually in the estimation of e.s.d.'s in distances, angles and torsion angles; correlations between e.s.d.'s in cell parameters are only used when they are defined by crystal symmetry. An approximate (isotropic) treatment of cell e.s.d.'s is used for estimating e.s.d.'s involving l.s. planes.Least-squares planes (*x*,*y*,*z* in crystal coordinates) and deviations from them (* indicates atom used to define plane)13.0283 (0.0011) *x* + 1.4133 (0.0037) *y* - 6.8581 (0.0016) *z* = 2.4289 (0.0026)* 0.0169 (0.0013) C6 * 0.0162 (0.0012) C7 * 0.0312 (0.0012) C8 * 0.0483 (0.0013) C9 * 0.0676 (0.0014) C10 * 0.0481 (0.0013) C11 * 0.0436 (0.0010) O2 * -0.0206 (0.0014) C12 * -0.1392 (0.0013) C13 * 0.0011 (0.0013) C5 * -0.0780 (0.0010) O1 * 0.0952 (0.0010) N4 * -0.1306 (0.0010) N3Rms deviation of fitted atoms = 0.0705- 5.8835 (0.0067) *x* + 8.3025 (0.0025) *y* + 4.3848 (0.0043) *z* = 4.5767 (0.0054)Angle to previous plane (with approximate e.s.d.) = 68.09 (0.03)* -0.0909 (0.0013) N1 * -0.0296 (0.0012) C2 * 0.0247 (0.0006) S * 0.0499 (0.0008) N4 * -0.0143 (0.0011) N3 * 0.0602 (0.0008) C14Rms deviation of fitted atoms = 0.05184.4124 (0.0064) *x* - 7.2670 (0.0036) *y* + 6.6294 (0.0075) *z* = 4.1549 (0.0063)Angle to previous plane (with approximate e.s.d.) = 56.56 (0.04)* 0.0131 (0.0015) C14 * 0.0077 (0.0017) C15 * -0.0154 (0.0018) C16 * 0.0007 (0.0017) C17 * -0.0065 (0.0015) C18 * -0.0105 (0.0015) C19 * -0.0002 (0.0013) O3 * 0.0099 (0.0013) C20 * 0.0012 (0.0012) N1Rms deviation of fitted atoms = 0.008913.0659 (0.0012) *x* + 1.8489 (0.0037) *y* - 6.6371 (0.0017) *z* = 6.1077 (0.0017)Angle to previous plane (with approximate e.s.d.) = 78.79 (0.03)* 0.0172 (0.0013) C6' * 0.0319 (0.0011) C7' * 0.0585 (0.0011) C8' * 0.0569 (0.0013) C9' * 0.0548 (0.0014) C10' * 0.0249 (0.0013) C11' * 0.0496 (0.0010) O2' * -0.0083 (0.0015) C12' * -0.1597 (0.0014) C13' * -0.0114 (0.0013) C5' * -0.1233 (0.0010) O1' * 0.1239 (0.0010) N4' * -0.1150 (0.0010) N3'Rms deviation of fitted atoms = 0.0802- 4.7715 (0.0070) *x* + 8.8203 (0.0020) *y* + 3.4544 (0.0044) *z* = 2.9970 (0.0051)Angle to previous plane (with approximate e.s.d.) = 77.22 (0.03)* -0.0881 (0.0013) N1' * -0.0271 (0.0012) C2' * 0.0344 (0.0006) S' * 0.0675 (0.0008) N4' * -0.0418 (0.0011) N3' * 0.0550 (0.0008) C14'Rms deviation of fitted atoms = 0.05632.1176 (0.0060) *x* - 6.0315 (0.0044) *y* + 8.8245 (0.0052) *z* = 0.7518 (0.0041)Angle to previous plane (with approximate e.s.d.) = 66.14 (0.03)* 0.0140 (0.0014) C14' * 0.0048 (0.0014) C15' * -0.0082 (0.0014) C16' * -0.0043 (0.0016) C17' * -0.0002 (0.0015) C18' * -0.0089 (0.0014) C19' * -0.0003 (0.0013) O3' * 0.0060 (0.0013) C20' * -0.0029 (0.0012) N1'Rms deviation of fitted atoms = 0.0069
Refinement. Refinement of *F*^2^ against ALL reflections. The weighted *R*-factor *wR* and goodness of fit *S* are based on *F*^2^, conventional *R*-factors *R* are based on *F*, with *F* set to zero for negative *F*^2^. The threshold expression of *F*^2^ > σ(*F*^2^) is used only for calculating *R*-factors(gt) *etc*. and is not relevant to the choice of reflections for refinement. *R*-factors based on *F*^2^ are statistically about twice as large as those based on *F*, and *R*- factors based on ALL data will be even larger.

### Fractional atomic coordinates and isotropic or equivalent isotropic displacement parameters (Å^2^)

**Table:**

	*x*	*y*	*z*	*U*_iso_*/*U*_eq_	
S	0.70206 (2)	0.50456 (5)	1.03604 (3)	0.01728 (10)	
O1	0.52373 (7)	0.30901 (11)	0.71581 (8)	0.0180 (2)	
O2	0.28479 (6)	0.55263 (11)	0.29437 (8)	0.0211 (3)	
O3	1.02907 (7)	0.86352 (16)	0.88836 (10)	0.0411 (4)	
N1	0.70429 (8)	0.60538 (15)	0.82176 (11)	0.0181 (3)	
H01	0.6823 (9)	0.6052 (17)	0.7547 (10)	0.021 (5)*	
C2	0.66134 (9)	0.54043 (16)	0.90110 (11)	0.0125 (3)	
N3	0.57906 (8)	0.50134 (15)	0.86822 (10)	0.0142 (3)	
H03	0.5512 (8)	0.4637 (14)	0.9164 (10)	0.005 (4)*	
N4	0.53669 (8)	0.53524 (14)	0.76180 (10)	0.0133 (3)	
H04	0.5143 (10)	0.6113 (14)	0.7606 (14)	0.027 (6)*	
C5	0.50445 (9)	0.42990 (16)	0.69256 (13)	0.0128 (3)	
C6	0.44704 (9)	0.47015 (16)	0.58951 (12)	0.0122 (3)	
C7	0.41778 (9)	0.36550 (16)	0.51243 (12)	0.0148 (4)	
H7	0.4349	0.2724	0.5274	0.018*	
C8	0.36421 (10)	0.39690 (17)	0.41495 (12)	0.0169 (4)	
H8	0.3447	0.3252	0.3634	0.020*	
C9	0.33859 (9)	0.53260 (17)	0.39175 (12)	0.0157 (3)	
C10	0.36805 (10)	0.63793 (17)	0.46663 (12)	0.0171 (4)	
H10	0.3516	0.7311	0.4507	0.021*	
C11	0.42156 (10)	0.60574 (16)	0.56447 (13)	0.0157 (4)	
H11	0.4413	0.6777	0.6156	0.019*	
C12	0.25273 (10)	0.69102 (17)	0.27136 (13)	0.0244 (4)	
H12A	0.3015	0.7539	0.2577	0.029*	
H12B	0.2234	0.7264	0.3374	0.029*	
C13	0.18893 (11)	0.68298 (19)	0.16578 (13)	0.0312 (5)	
H13A	0.2177	0.6421	0.1025	0.047*	
H13B	0.1685	0.7762	0.1443	0.047*	
H13C	0.1391	0.6255	0.1821	0.047*	
C14	0.78727 (10)	0.67202 (18)	0.84138 (13)	0.0198 (4)	
C15	0.85155 (11)	0.6460 (2)	0.76923 (14)	0.0428 (6)	
H15	0.8414	0.5821	0.7078	0.051*	
C16	0.93107 (12)	0.7134 (3)	0.78670 (14)	0.0503 (7)	
H16	0.9746	0.6970	0.7357	0.060*	
C17	0.94779 (10)	0.8035 (2)	0.87684 (14)	0.0298 (5)	
C18	0.88347 (10)	0.83019 (18)	0.94777 (15)	0.0270 (4)	
H18	0.8938	0.8933	1.0097	0.032*	
C19	0.80302 (10)	0.76466 (18)	0.92888 (15)	0.0272 (4)	
H19	0.7584	0.7846	0.9775	0.033*	
C20	1.04658 (11)	0.9572 (2)	0.98097 (15)	0.0376 (5)	
H20A	1.0051	1.0342	0.9729	0.056*	
H20B	1.1061	0.9930	0.9803	0.056*	
H20C	1.0406	0.9092	1.0536	0.056*	
S'	0.69682 (2)	0.51034 (4)	0.53698 (3)	0.01624 (9)	
O1'	0.52337 (7)	0.30780 (11)	0.21440 (8)	0.0184 (2)	
O2'	0.28670 (6)	0.54492 (11)	−0.21151 (8)	0.0215 (3)	
O3'	1.04034 (7)	0.80274 (17)	0.38417 (11)	0.0439 (4)	
N1'	0.70424 (8)	0.58667 (14)	0.31685 (11)	0.0161 (3)	
H01'	0.6834 (9)	0.5828 (16)	0.2504 (10)	0.017 (5)*	
C2'	0.65809 (9)	0.53631 (15)	0.39935 (11)	0.0122 (3)	
N3'	0.57447 (7)	0.50178 (15)	0.36778 (10)	0.0139 (3)	
H03'	0.5419 (8)	0.4738 (15)	0.4176 (10)	0.012 (4)*	
N4'	0.53359 (8)	0.53414 (14)	0.26033 (10)	0.0133 (3)	
H04'	0.5104 (10)	0.6123 (14)	0.2551 (13)	0.023 (5)*	
C5'	0.50319 (9)	0.42750 (16)	0.19115 (12)	0.0130 (3)	
C6'	0.44667 (9)	0.46655 (16)	0.08647 (12)	0.0118 (3)	
C7'	0.42218 (9)	0.36192 (16)	0.00689 (12)	0.0140 (3)	
H7'	0.4418	0.2699	0.0212	0.017*	
C8'	0.36966 (9)	0.39163 (17)	−0.09224 (12)	0.0152 (3)	
H8'	0.3539	0.3203	−0.1460	0.018*	
C9'	0.33984 (9)	0.52599 (17)	−0.11327 (12)	0.0157 (3)	
C10'	0.36423 (10)	0.63083 (17)	−0.03574 (13)	0.0173 (4)	
H10'	0.3450	0.7229	−0.0508	0.021*	
C11'	0.41673 (10)	0.60055 (16)	0.06368 (13)	0.0155 (4)	
H11'	0.4325	0.6723	0.1170	0.019*	
C12'	0.25161 (11)	0.68144 (18)	−0.23382 (14)	0.0274 (4)	
H12C	0.2990	0.7471	−0.2472	0.033*	
H12D	0.2217	0.7144	−0.1675	0.033*	
C13'	0.18795 (12)	0.6718 (2)	−0.33901 (14)	0.0392 (5)	
H13D	0.2167	0.6298	−0.4019	0.059*	
H13E	0.1676	0.7647	−0.3616	0.059*	
H13F	0.1381	0.6147	−0.3220	0.059*	
C14'	0.79061 (10)	0.64214 (18)	0.33595 (13)	0.0187 (4)	
C15'	0.85761 (10)	0.5858 (2)	0.28031 (13)	0.0280 (4)	
H15'	0.8472	0.5088	0.2305	0.034*	
C16'	0.94058 (10)	0.6430 (2)	0.29804 (13)	0.0350 (5)	
H16'	0.9869	0.6051	0.2597	0.042*	
C17'	0.95622 (10)	0.7544 (2)	0.37088 (14)	0.0294 (5)	
C18'	0.88890 (10)	0.81103 (19)	0.42619 (15)	0.0282 (4)	
H18'	0.8994	0.8873	0.4767	0.034*	
C19'	0.80553 (10)	0.75494 (18)	0.40688 (14)	0.0259 (4)	
H19'	0.7586	0.7948	0.4429	0.031*	
C20'	1.05700 (12)	0.9175 (2)	0.45930 (16)	0.0441 (6)	
H20D	1.0241	0.9980	0.4285	0.066*	
H20E	1.1194	0.9387	0.4658	0.066*	
H20F	1.0389	0.8946	0.5352	0.066*	

### Atomic displacement parameters (Å^2^)

**Table:**

	*U*^11^	*U*^22^	*U*^33^	*U*^12^	*U*^13^	*U*^23^
S	0.0191 (2)	0.0208 (3)	0.01124 (19)	−0.0005 (2)	−0.00234 (16)	0.00053 (18)
O1	0.0241 (6)	0.0118 (6)	0.0177 (6)	0.0013 (5)	−0.0002 (5)	0.0031 (5)
O2	0.0243 (6)	0.0218 (7)	0.0157 (6)	0.0006 (5)	−0.0066 (5)	0.0002 (5)
O3	0.0235 (7)	0.0669 (11)	0.0330 (7)	−0.0240 (7)	0.0037 (6)	−0.0058 (7)
N1	0.0150 (7)	0.0272 (9)	0.0117 (7)	−0.0055 (6)	−0.0012 (6)	0.0051 (6)
C2	0.0115 (7)	0.0120 (9)	0.0140 (8)	0.0012 (6)	0.0010 (6)	−0.0018 (6)
N3	0.0154 (6)	0.0180 (8)	0.0092 (7)	−0.0034 (6)	0.0009 (5)	0.0031 (6)
N4	0.0148 (7)	0.0116 (8)	0.0127 (7)	0.0000 (6)	−0.0026 (5)	0.0001 (6)
C5	0.0123 (8)	0.0125 (9)	0.0143 (8)	−0.0011 (7)	0.0043 (6)	0.0009 (7)
C6	0.0129 (8)	0.0110 (9)	0.0131 (8)	−0.0026 (6)	0.0023 (6)	−0.0001 (6)
C7	0.0164 (8)	0.0111 (9)	0.0172 (9)	−0.0009 (7)	0.0039 (7)	0.0010 (7)
C8	0.0177 (8)	0.0182 (10)	0.0144 (8)	−0.0043 (7)	−0.0002 (7)	−0.0049 (7)
C9	0.0121 (7)	0.0210 (10)	0.0138 (8)	−0.0019 (7)	0.0004 (6)	0.0034 (7)
C10	0.0221 (8)	0.0117 (9)	0.0170 (9)	0.0005 (7)	−0.0009 (7)	−0.0008 (7)
C11	0.0174 (8)	0.0131 (10)	0.0161 (9)	−0.0017 (7)	−0.0016 (7)	−0.0051 (7)
C12	0.0244 (10)	0.0245 (11)	0.0233 (10)	0.0044 (8)	−0.0041 (8)	0.0023 (8)
C13	0.0273 (10)	0.0399 (12)	0.0246 (10)	0.0040 (8)	−0.0076 (8)	0.0047 (8)
C14	0.0119 (8)	0.0278 (10)	0.0194 (9)	−0.0038 (7)	−0.0005 (6)	0.0082 (7)
C15	0.0288 (10)	0.0805 (18)	0.0202 (9)	−0.0255 (11)	0.0082 (7)	−0.0192 (10)
C16	0.0298 (11)	0.096 (2)	0.0274 (10)	−0.0292 (11)	0.0170 (8)	−0.0178 (11)
C17	0.0194 (9)	0.0454 (13)	0.0240 (9)	−0.0128 (8)	−0.0023 (7)	0.0086 (8)
C18	0.0211 (9)	0.0215 (10)	0.0387 (10)	−0.0053 (7)	0.0048 (8)	−0.0080 (8)
C19	0.0180 (9)	0.0214 (10)	0.0434 (10)	−0.0038 (7)	0.0087 (8)	−0.0077 (8)
C20	0.0245 (9)	0.0496 (13)	0.0374 (10)	−0.0197 (9)	−0.0049 (8)	0.0030 (9)
S'	0.0178 (2)	0.0181 (2)	0.01218 (19)	0.0001 (2)	−0.00225 (15)	−0.00011 (18)
O1'	0.0255 (6)	0.0116 (7)	0.0178 (6)	0.0013 (5)	−0.0007 (5)	0.0025 (5)
O2'	0.0246 (6)	0.0232 (7)	0.0151 (6)	−0.0011 (5)	−0.0079 (5)	−0.0006 (5)
O3'	0.0182 (6)	0.0690 (11)	0.0448 (8)	−0.0180 (7)	0.0041 (6)	−0.0062 (7)
N1'	0.0131 (7)	0.0236 (8)	0.0113 (7)	−0.0032 (6)	−0.0004 (6)	0.0006 (6)
C2'	0.0149 (8)	0.0081 (9)	0.0139 (8)	0.0017 (6)	0.0026 (6)	−0.0011 (6)
N3'	0.0121 (6)	0.0188 (8)	0.0105 (7)	−0.0020 (6)	−0.0004 (5)	0.0015 (6)
N4'	0.0139 (7)	0.0130 (9)	0.0124 (7)	0.0014 (6)	−0.0019 (5)	0.0020 (6)
C5'	0.0128 (8)	0.0138 (9)	0.0131 (8)	−0.0018 (7)	0.0042 (6)	0.0003 (7)
C6'	0.0096 (7)	0.0125 (9)	0.0134 (8)	−0.0018 (6)	0.0019 (6)	0.0016 (7)
C7'	0.0154 (8)	0.0114 (9)	0.0156 (8)	−0.0026 (7)	0.0037 (7)	−0.0018 (7)
C8'	0.0155 (8)	0.0156 (9)	0.0148 (8)	−0.0047 (7)	0.0031 (6)	−0.0026 (7)
C9'	0.0130 (7)	0.0238 (10)	0.0103 (7)	−0.0012 (7)	0.0006 (6)	0.0009 (7)
C10'	0.0180 (8)	0.0158 (9)	0.0178 (9)	0.0039 (7)	−0.0008 (7)	0.0037 (7)
C11'	0.0185 (8)	0.0131 (9)	0.0149 (9)	−0.0012 (7)	0.0008 (7)	−0.0017 (7)
C12'	0.0286 (10)	0.0264 (11)	0.0255 (10)	0.0024 (8)	−0.0079 (8)	0.0030 (8)
C13'	0.0399 (12)	0.0448 (14)	0.0299 (11)	0.0070 (10)	−0.0142 (9)	0.0026 (9)
C14'	0.0142 (8)	0.0253 (10)	0.0165 (8)	−0.0027 (7)	0.0009 (6)	0.0047 (7)
C15'	0.0187 (8)	0.0423 (12)	0.0233 (9)	−0.0025 (8)	0.0040 (7)	−0.0088 (8)
C16'	0.0164 (9)	0.0614 (14)	0.0277 (10)	−0.0018 (9)	0.0056 (7)	−0.0101 (9)
C17'	0.0150 (8)	0.0459 (13)	0.0266 (9)	−0.0101 (8)	−0.0013 (7)	0.0057 (9)
C18'	0.0212 (9)	0.0248 (10)	0.0384 (10)	−0.0065 (8)	0.0009 (8)	−0.0009 (8)
C19'	0.0145 (8)	0.0262 (11)	0.0377 (10)	−0.0007 (7)	0.0060 (7)	−0.0030 (8)
C20'	0.0260 (10)	0.0522 (14)	0.0521 (13)	−0.0214 (10)	−0.0083 (9)	0.0102 (10)

### Geometric parameters (Å, °)

**Table:**

S—C2	1.6823 (14)	C14'—C19'	1.378 (2)
O1—C5	1.2307 (17)	C14'—C15'	1.380 (2)
O2—C9	1.3618 (16)	C15'—C16'	1.391 (2)
O2—C12	1.4436 (18)	C16'—C17'	1.382 (2)
O3—C17	1.3757 (18)	C17'—C18'	1.383 (2)
O3—C20	1.420 (2)	C18'—C19'	1.394 (2)
N1—C2	1.3428 (18)	N1—H01	0.826 (11)
N1—C14	1.4316 (19)	N3—H03	0.822 (10)
C2—N3	1.3457 (17)	N4—H04	0.812 (12)
N3—N4	1.3926 (16)	C7—H7	0.9500
N4—C5	1.3672 (19)	C8—H8	0.9500
C5—C6	1.482 (2)	C10—H10	0.9500
C6—C11	1.393 (2)	C11—H11	0.9500
C6—C7	1.4030 (19)	C12—H12A	0.9900
C7—C8	1.3809 (19)	C12—H12B	0.9900
C8—C9	1.390 (2)	C13—H13A	0.9800
C9—C10	1.393 (2)	C13—H13B	0.9800
C10—C11	1.3853 (19)	C13—H13C	0.9800
C12—C13	1.5108 (19)	C15—H15	0.9500
C14—C19	1.366 (2)	C16—H16	0.9500
C14—C15	1.380 (2)	C18—H18	0.9500
C15—C16	1.387 (2)	C19—H19	0.9500
C16—C17	1.375 (3)	C20—H20A	0.9800
C17—C18	1.372 (2)	C20—H20B	0.9800
C18—C19	1.392 (2)	C20—H20C	0.9800
S'—C2'	1.6856 (14)	N1'—H01'	0.816 (11)
O1'—C5'	1.2229 (17)	N3'—H03'	0.847 (10)
O2'—C9'	1.3630 (16)	N4'—H04'	0.836 (12)
O2'—C12'	1.4417 (19)	C7'—H7'	0.9500
O3'—C17'	1.3733 (18)	C8'—H8'	0.9500
O3'—C20'	1.425 (2)	C10'—H10'	0.9500
N1'—C2'	1.3402 (17)	C11'—H11'	0.9500
N1'—C14'	1.4335 (19)	C12'—H12C	0.9900
C2'—N3'	1.3501 (17)	C12'—H12D	0.9900
N3'—N4'	1.3908 (16)	C13'—H13D	0.9800
N4'—C5'	1.3678 (19)	C13'—H13E	0.9800
C5'—C6'	1.487 (2)	C13'—H13F	0.9800
C6'—C11'	1.393 (2)	C15'—H15'	0.9500
C6'—C7'	1.4039 (19)	C16'—H16'	0.9500
C7'—C8'	1.3840 (19)	C18'—H18'	0.9500
C8'—C9'	1.393 (2)	C19'—H19'	0.9500
C9'—C10'	1.390 (2)	C20'—H20D	0.9800
C10'—C11'	1.3877 (19)	C20'—H20E	0.9800
C12'—C13'	1.507 (2)	C20'—H20F	0.9800
			
C9—O2—C12	117.42 (12)	C6—C7—H7	119.8
C17—O3—C20	116.97 (14)	C7—C8—H8	119.8
C2—N1—C14	125.98 (13)	C9—C8—H8	119.8
N1—C2—N3	116.31 (13)	C11—C10—H10	120.3
N1—C2—S	125.42 (11)	C9—C10—H10	120.3
N3—C2—S	118.27 (11)	C10—C11—H11	119.3
C2—N3—N4	122.97 (12)	C6—C11—H11	119.3
C5—N4—N3	118.10 (13)	O2—C12—H12A	110.3
O1—C5—N4	120.60 (15)	C13—C12—H12A	110.3
O1—C5—C6	122.96 (14)	O2—C12—H12B	110.3
N4—C5—C6	116.44 (14)	C13—C12—H12B	110.3
C11—C6—C7	118.40 (14)	H12A—C12—H12B	108.6
C11—C6—C5	123.71 (13)	C12—C13—H13A	109.5
C7—C6—C5	117.89 (14)	C12—C13—H13B	109.5
C8—C7—C6	120.43 (15)	H13A—C13—H13B	109.5
C7—C8—C9	120.49 (15)	C12—C13—H13C	109.5
O2—C9—C8	116.02 (14)	H13A—C13—H13C	109.5
O2—C9—C10	124.21 (15)	H13B—C13—H13C	109.5
C8—C9—C10	119.77 (14)	C14—C15—H15	120.1
C11—C10—C9	119.48 (15)	C16—C15—H15	120.1
C10—C11—C6	121.42 (14)	C17—C16—H16	119.5
O2—C12—C13	106.96 (13)	C15—C16—H16	119.5
C19—C14—C15	119.26 (15)	C17—C18—H18	120.0
C19—C14—N1	120.81 (15)	C19—C18—H18	120.0
C15—C14—N1	119.90 (15)	C14—C19—H19	119.5
C14—C15—C16	119.76 (17)	C18—C19—H19	119.5
C17—C16—C15	120.94 (17)	O3—C20—H20A	109.5
C18—C17—C16	119.13 (16)	O3—C20—H20B	109.5
C18—C17—O3	124.15 (16)	H20A—C20—H20B	109.5
C16—C17—O3	116.71 (16)	O3—C20—H20C	109.5
C17—C18—C19	119.93 (16)	H20A—C20—H20C	109.5
C14—C19—C18	120.94 (16)	H20B—C20—H20C	109.5
C9'—O2'—C12'	117.59 (12)	C2'—N1'—H01'	118.7 (11)
C17'—O3'—C20'	116.84 (14)	C14'—N1'—H01'	116.6 (11)
C2'—N1'—C14'	124.62 (13)	C2'—N3'—H03'	120.0 (9)
N1'—C2'—N3'	116.56 (13)	N4'—N3'—H03'	116.5 (9)
N1'—C2'—S'	125.22 (11)	C5'—N4'—H04'	121.1 (11)
N3'—C2'—S'	118.21 (11)	N3'—N4'—H04'	115.2 (11)
C2'—N3'—N4'	122.41 (12)	C8'—C7'—H7'	119.7
C5'—N4'—N3'	117.96 (13)	C6'—C7'—H7'	119.7
O1'—C5'—N4'	121.01 (15)	C7'—C8'—H8'	120.0
O1'—C5'—C6'	122.84 (14)	C9'—C8'—H8'	120.0
N4'—C5'—C6'	116.14 (14)	C11'—C10'—H10'	120.1
C11'—C6'—C7'	118.59 (14)	C9'—C10'—H10'	120.1
C11'—C6'—C5'	123.61 (14)	C10'—C11'—H11'	119.5
C7'—C6'—C5'	117.80 (14)	C6'—C11'—H11'	119.5
C8'—C7'—C6'	120.58 (15)	O2'—C12'—H12C	110.3
C7'—C8'—C9'	120.05 (14)	C13'—C12'—H12C	110.3
O2'—C9'—C10'	124.03 (15)	O2'—C12'—H12D	110.3
O2'—C9'—C8'	115.99 (14)	C13'—C12'—H12D	110.3
C10'—C9'—C8'	119.97 (14)	H12C—C12'—H12D	108.5
C11'—C10'—C9'	119.79 (15)	C12'—C13'—H13D	109.5
C10'—C11'—C6'	121.00 (15)	C12'—C13'—H13E	109.5
O2'—C12'—C13'	107.32 (14)	H13D—C13'—H13E	109.5
C19'—C14'—C15'	120.33 (15)	C12'—C13'—H13F	109.5
C19'—C14'—N1'	119.55 (15)	H13D—C13'—H13F	109.5
C15'—C14'—N1'	120.08 (15)	H13E—C13'—H13F	109.5
C14'—C15'—C16'	119.21 (17)	C14'—C15'—H15'	120.4
C17'—C16'—C15'	120.65 (16)	C16'—C15'—H15'	120.4
O3'—C17'—C16'	116.52 (16)	C17'—C16'—H16'	119.7
O3'—C17'—C18'	123.46 (17)	C15'—C16'—H16'	119.7
C16'—C17'—C18'	120.02 (16)	C17'—C18'—H18'	120.4
C17'—C18'—C19'	119.23 (17)	C19'—C18'—H18'	120.4
C14'—C19'—C18'	120.52 (16)	C14'—C19'—H19'	119.7
C2—N1—H01	118.0 (11)	C18'—C19'—H19'	119.7
C14—N1—H01	116.0 (11)	O3'—C20'—H20D	109.5
C2—N3—H03	117.6 (9)	O3'—C20'—H20E	109.5
N4—N3—H03	119.0 (9)	H20D—C20'—H20E	109.5
C5—N4—H04	122.2 (12)	O3'—C20'—H20F	109.5
N3—N4—H04	113.2 (12)	H20D—C20'—H20F	109.5
C8—C7—H7	119.8	H20E—C20'—H20F	109.5
			
C14—N1—C2—N3	171.14 (15)	C14'—N1'—C2'—N3'	173.21 (15)
C14—N1—C2—S	−9.0 (2)	C14'—N1'—C2'—S'	−8.1 (2)
N1—C2—N3—N4	−5.3 (2)	N1'—C2'—N3'—N4'	−9.0 (2)
S—C2—N3—N4	174.82 (11)	S'—C2'—N3'—N4'	172.22 (11)
C2—N3—N4—C5	123.25 (16)	C2'—N3'—N4'—C5'	119.34 (16)
N3—N4—C5—O1	−10.8 (2)	N3'—N4'—C5'—O1'	−11.7 (2)
N3—N4—C5—C6	169.88 (12)	N3'—N4'—C5'—C6'	169.66 (11)
O1—C5—C6—C11	177.14 (15)	O1'—C5'—C6'—C11'	174.11 (15)
N4—C5—C6—C11	−3.6 (2)	N4'—C5'—C6'—C11'	−7.2 (2)
O1—C5—C6—C7	−3.6 (2)	O1'—C5'—C6'—C7'	−5.6 (2)
N4—C5—C6—C7	175.64 (13)	N4'—C5'—C6'—C7'	173.03 (13)
C11—C6—C7—C8	−0.7 (2)	C11'—C6'—C7'—C8'	0.2 (2)
C5—C6—C7—C8	−179.97 (13)	C5'—C6'—C7'—C8'	180.00 (13)
C6—C7—C8—C9	0.1 (2)	C6'—C7'—C8'—C9'	−0.7 (2)
C12—O2—C9—C8	176.35 (13)	C12'—O2'—C9'—C10'	−2.1 (2)
C12—O2—C9—C10	−3.6 (2)	C12'—O2'—C9'—C8'	177.49 (13)
C7—C8—C9—O2	−179.13 (12)	C7'—C8'—C9'—O2'	−178.38 (12)
C7—C8—C9—C10	0.8 (2)	C7'—C8'—C9'—C10'	1.2 (2)
O2—C9—C10—C11	178.93 (13)	O2'—C9'—C10'—C11'	178.20 (13)
C8—C9—C10—C11	−1.0 (2)	C8'—C9'—C10'—C11'	−1.4 (2)
C9—C10—C11—C6	0.4 (2)	C9'—C10'—C11'—C6'	1.0 (2)
C7—C6—C11—C10	0.5 (2)	C7'—C6'—C11'—C10'	−0.4 (2)
C5—C6—C11—C10	179.72 (13)	C5'—C6'—C11'—C10'	179.86 (13)
C9—O2—C12—C13	−174.97 (12)	C9'—O2'—C12'—C13'	−173.82 (13)
C2—N1—C14—C19	−50.0 (2)	C2'—N1'—C14'—C19'	−60.2 (2)
C2—N1—C14—C15	131.93 (18)	C2'—N1'—C14'—C15'	122.06 (18)
C19—C14—C15—C16	0.3 (3)	C19'—C14'—C15'—C16'	0.9 (3)
N1—C14—C15—C16	178.35 (18)	N1'—C14'—C15'—C16'	178.60 (16)
C14—C15—C16—C17	1.6 (3)	C14'—C15'—C16'—C17'	0.4 (3)
C15—C16—C17—C18	−2.2 (3)	C20'—O3'—C17'—C16'	−179.90 (16)
C15—C16—C17—O3	178.87 (19)	C20'—O3'—C17'—C18'	−0.1 (3)
C20—O3—C17—C18	0.9 (3)	C15'—C16'—C17'—O3'	179.18 (16)
C20—O3—C17—C16	179.71 (18)	C15'—C16'—C17'—C18'	−0.6 (3)
C16—C17—C18—C19	0.9 (3)	O3'—C17'—C18'—C19'	179.81 (17)
O3—C17—C18—C19	179.73 (17)	C16'—C17'—C18'—C19'	−0.4 (3)
C15—C14—C19—C18	−1.6 (3)	C15'—C14'—C19'—C18'	−2.0 (3)
N1—C14—C19—C18	−179.64 (16)	N1'—C14'—C19'—C18'	−179.66 (15)
C17—C18—C19—C14	1.0 (3)	C17'—C18'—C19'—C14'	1.7 (3)

### Hydrogen-bond geometry (Å, °)

**Table:**

*D*—H···*A*	*D*—H	H···*A*	*D*···*A*	*D*—H···*A*
N1—H01···S'	0.83 (1)	2.74 (1)	3.4483 (14)	146 (1)
N4—H04···O1'^i^	0.81 (1)	2.02 (1)	2.8156 (17)	168 (2)
N1'—H01'···S^ii^	0.82 (1)	2.66 (1)	3.3772 (14)	148 (1)
N4'—H04'···O1^i^	0.84 (1)	2.01 (1)	2.8228 (18)	164 (2)
C11'—H11'···O1^i^	0.95	2.41	3.3378 (19)	166
C11—H11···O1'^i^	0.95	2.38	3.2927 (18)	162
C20—H20B···S^iii^	0.98	2.98	3.9215 (17)	161
C20'—H20E···S'^iv^	0.98	2.92	3.8936 (18)	175
C10'—H10'···S^i^	0.95	2.83	3.7559 (17)	166
C10—H10···S'^i^	0.95	2.81	3.7369 (17)	166
C15'—H15'···S^ii^	0.95	3.05	3.6482 (16)	123
C15—H15···S'	0.95	2.94	3.6925 (17)	137
N3'—H03'···Cg	0.85 (1)	2.53	3.25	144
N3—H03···Cg'^v^	0.82 (1)	2.65	3.29	135

Symmetry codes: (i) −*x*+1, *y*+1/2, −*z*+1; (ii) *x*, *y*, *z*−1; (iii) −*x*+2, *y*+1/2, −*z*+2; (iv) −*x*+2, *y*+1/2, −*z*+1; (v) *x*, *y*, *z*+1.
